# The impact of DXA findings on general practitioners’ decision regarding first-line oral bisphosphonates in postmenopausal osteoporosis—data from French General Practice (the IMOGENE study)

**DOI:** 10.1007/s11657-026-01663-3

**Published:** 2026-02-13

**Authors:** Bernard Cortet, Leila Chelbani, Térésa Garot, Pascale Samama, Eric Lespessailles

**Affiliations:** 1 Rheumatology Department, University Hospital, Lille, France; 2 Amgen SAS, 20 Quai du Point du Jour, CS 10096, 92650 Boulogne Billancourt Cedex, France; 3 ICTA PM, 11 Rue du Bocage, 21121 Dijon, France; 4 Rheumatology Department, University Hospital, Orleans, France

**Keywords:** Postmenopausal osteoporosis (PMO), Oral bisphosphonates, Bone mineral density (BMD), General practitioners (GP), guidelines, Dual energy X-ray absorptiometry (DXA)

## Abstract

***Summary*:**

In our real-world study of women with postmenopausal osteoporosis (PMO), general practitioners (GPs) adhered to French guidelines on stopping, changing, or continuing oral bisphosphonates in 60% of cases and adhered to guidelines on initiating oral BPs in only 39%. These findings highlight the need to educate GPs on PMO treatment recommendations.

**Purpose:**

French guidelines for postmenopausal osteoporosis (PMO) recommend dual energy X-ray absorptiometry (DXA) assessment of bone mineral density (BMD) to guide treatment decisions. However, data reporting implementation of these guidelines in general practice is lacking. Our study assessed general practitioners’ (GPs’) use of DXA results to guide decisions regarding first-line oral bisphosphonate (oBP) treatment in women with PMO.

**Methods:**

In this multicenter cohort study, participating GPs enrolled women with PMO who had been receiving the first-line bisphosphonates for 2–5 years, had a reference (baseline) DXA in the 2 years pre-enrolment or ≤ 2 years prior to BP initiation, and agreed to a follow-up DXA. GPs prescribed a follow-up DXA to guide their decision to stop, continue, or change BPs. We checked the GPs’ decision for concordance with national treatment guidelines.

**Results:**

From January 2018 to November 2019, 23 GPs enrolled 99 women meeting the inclusion criteria. Based on follow-up DXA, the decision to stop, change, or continue oBPs aligned with guidelines in 60% of cases. Agreement was higher in women receiving oBPs for < 3 vs ≥ 3 years (70.2% vs 54.3%). Based on baseline DXA, the decision to initiate treatment was aligned in only 39% of cases, with the follow-up treatment decision aligned with guidelines in 72% of these cases. The consistency of treatment decision between GPs and the scientific committee was weak (kappa coefficient of 0.295).

**Conclusion:**

Our study suggests insufficient awareness of national recommendations for PMO treatment in French general practice, highlighting the need for stronger GP education.

## Introduction

Osteoporosis affects approximately one in three women and one in five men aged over 50 years worldwide [1]. Thus, postmenopausal osteoporosis (PMO) poses a significant health concern in women, placing them at high risk of low-impact, fragility fractures. Fragility fractures most commonly occur at the hip, wrist, and vertebrae [2], with the hip being the most prevalent fracture site and associated with a 25% reduction in life expectancy and substantially impaired quality of life [3, 4].

Bone densitometry, commonly measured by Dual-energy X-ray Absorptiometry (DXA), is the gold standard for diagnosing PMO in clinical practice. Specifically, hip or spine bone mineral density (BMD) measured by DXA is used to evaluate fracture risk and guide treatment decisions [5, 6]. Bisphosphonates (BPs; in most cases oral) are the most common first-line treatment for PMO, with treatment duration dependent on several factors [7]. Considering the prolonged presence of BPs in bone and the increased risk of rare adverse events with their long-term use, published guidelines for the management of osteoporosis suggest reevaluating whether to continue BPs after 3 to 5 years [8, 9]. National guidelines published by the French Society of Rheumatology and Osteoporosis Research and Information Group (GRIO) [5, 7] advise reevaluating BP treatment 2 to 3 years after initiation using BMD measured by DXA, with this time interval considered suitable to identify significant changes in BMD attributable to treatment. For example, regardless of the location, a BMD loss > 0.03 g/cm^2^ 2 to 3 years after treatment initiation should lead to a re-evaluation of the treatment [5]. In addition, treatment discontinuation is recommended if the following conditions are met after 3 to 5 years of BP treatment: no fractures while on treatment, no new risk factors, no significant decrease in BMD, and a *T*-score measured at the femoral neck > −2.5 (or > −2 standard deviations) [5].

Despite national guidelines, disparities in the management of osteoporosis are frequently observed in French clinical practice, and data regarding the follow-up of anti-osteoporotic treatments for patients with PMO in primary care in France is sparse [7]. Osteoporosis is a very frequent disease, and the majority of anti-osteoporotic treatments are prescribed by GPs. Our study evaluated general practitioner (GP) adherence to the French recommendations in women with PMO receiving first-line oral BP treatment for 2–5 years; specifically, whether GP decisions to initiate, continue, or change treatment aligned with SFR/GRIO guidelines.

## Methods

### Study design

IMOGENE (IMpact of a bone mineral density measure by Osteo-densitometry on treatment decision for post-menopausal osteoporotic women treated with bisphosphonates in GENEral practice) was a multicenter, interventional, cohort study conducted between 25 January 2018 and 30 November 2019.

The first step was to send an e-mail to the potential GP participants in which we indicated that the study aimed to study how GPs were managing patients with postmenopausal osteoporosis treated by bisphosphonates by mouth (alendronate or risedronate) for 2 to 5 years. We also indicated that we wanted to focus on the GP population because they are the more frequent prescribers of antiosteoporosis treatments. We chose oral bisphosphonates because they are the most prescribed, particularly in general practice. Moreover, in the e-mail, we indicated that the obtained results will be compared to French recommendations regarding the management of postmenopausal osteoporosis. However, we chose to not send the recommendations to avoid biases, and we wanted the answers from the GPs regarding the management to be spontaneous and not driven by the reading of the recommendations.

**Fig. 1:**
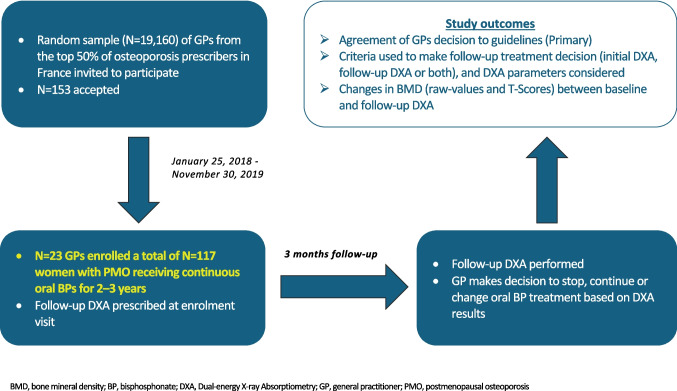
Study design

The study schema is shown in Fig. 1. At the enrolment visit, participating GPs prescribed a follow-up DXA to guide subsequent treatment decisions regarding oral BPs. Patient-level data, taken from medical records, DXA reports, and a semi-directive interview, were entered into an electronic Case Report Form (e-CRF). This included the investigator’s decision to continue, change, or stop oral BP treatment at the patient’s follow-up visit. The first and senior authors (BC and EL), rheumatologists with PMO expertise, reviewed the patient-level data and decided whether oral BP treatment should be continued, stopped, or changed according to SFR/GRIO guidelines. The decision taken by the GPs depended on the initial DXA, follow-up DXA, or both DXA (see below) and also on clinical parameters. The information collected permitted the calculation of the FRAX score, but the experts did not know if they did. The reason for this choice is that GPs are not familiar with FRAX in France (and therefore do not use it). Similarly, TBS in France is not generally measured at the same time as BMD. This is why these two parameters were not explicitly included in the CRF. BC and EL did not participate in the assessment of the patient to avoid any bias. They only checked a posteriori that the decision taken by the GPs was in line (or not) with the French recommendations regarding the management of postmenopausal osteoporosis. Their decision was then compared to the GPs’ decision recorded in the e-CRF. Although not included in the protocol, the authors also assessed the initial treatment decision against treatment guidelines. Moreover, we studied whether the agreement varied according to the duration of treatment by oBP and the age of the patient included.

In a first step, the experts (BC and EL) analyzed each case separately regarding the agreement of the decision of GP to the recommendations. For a few patients, there was a disagreement between the two experts. In these cases, a second round was performed and the final decision was obtained by adjudication. Therefore, the level of concordance of the two experts was 100%.

The study was approved by the Ethic and Scientific Committee CPP Ouest V under the number 2017-A02629-44. Written informed consent was obtained from all participants.

### Study population

The study enrolled women with PMO who had been receiving the first-line oral BPs for 2 to 5 years without discontinuation, who had a reference DXA performed ≤ 2 years prior to oral BP initiation or ≥ 2 years before inclusion, and who agreed to have a follow-up DXA. Women with metabolic bone disease or demineralizing osteopathy other than PMO, who had been receiving glucocorticoid treatment for > 3 months, were excluded.

GPs were chosen from a database of all French GPs provided by IMS Health (now IQVIA). After ranking GPs by estimated prescribing level in osteoporosis (highest to lowest), a random sample was chosen from the top 50% of prescribers. The participating GP population was defined as those who enrolled at least one patient into the study.

### Study outcomes

The primary study outcome was the proportion of patients for whom the participating GP decision to continue, change, or stop oral BP treatment at the follow-up visit was aligned with SFR/GRIO treatment guidelines [8]; specifically, the percentage of concordant cases between participating GPs and the authors.

Secondary outcomes included the following:

- The criteria GPs used to make the follow-up treatment decision
  - Initial DXA, follow-up DXA or both DXAs
  - BMD parameter(s) used: *T*-score, *Z*-score, value (g/cm^2^)
  - Site(s) assessed: lumbar spine, total hip, femoral neck
- The follow-up treatment decision taken: continue, change or discontinue oral BPs.
- Changes in BMD (raw values and *T*-scores) between baseline and follow-up DXA, with BMD gain defined as an increase > 0.03 g/cm^2^, BMD loss defined as a decrease > 0.03 g/cm^2^ and stable BMD defined as an increase or decrease < 0.03 g/cm^2^

### Statistical analysis

All analyses were descriptive, and no statistical tests were performed. Data are reported as observed, with no imputation for missing data. Quantitative data were described by missing data, mean, standard deviation (SD), median, 1 st and 3rd quartiles, minimum, and maximum. Qualitative data were described by missing data, and the number and percentage of patients in each category.

Data were analyzed for all enrolled patients who met the eligibility criteria and had initial and follow-up DXA results (Full Analysis Set [FAS]). Patients with protocol deviations were excluded from the FAS.

Post hoc analyses summarized primary and secondary outcomes according to the participating GPs’ follow-up treatment decision (stop, continue, or change) and for the subgroup of patients whose initial treatment decision was aligned with treatment guidelines. Post-hoc analyses also used Fleiss’ kappa coefficient [10] to measure the concordance between the participating GPs and authors, with a value < 0.4 considered poor concordance.

All statistical analyses were performed using SAS® software version 9.2 or upper (SAS Institute, North Carolina, USA).

## Results

### Study investigators

Of 19,160 GPs invited to participate in the study, 153 accepted and 23 enrolled at least one patient. Most (17/23 [73.9%]) participating GPs were male; median (range) age was 59 (41 to 69) years and median years of clinical practice was 28 (10 to 39). Most participating GPs practiced alone (15/23 [65.2%]) with a median (range) of 4 (1 to 15) patients enrolled per participating GP.

### Study participants

Of 117 patients enrolled (January 25, 2018, to November 30, 2019), 99 (84.6%) met the eligibility criteria and were included in the FAS. The main reasons for exclusion were non-eligibility (*n* = 7), a selection criterion declared not met by the investigator (*n* = 6), at least one missing or unavailable DXA (*n* = 3), and loss to follow-up (*n* = 2).

Baseline characteristics of patients included in the FAS are summarized in Table 1. The most prevalent comorbidity was hypertension, being reported in over half (53 [53.5]) of all patients; fewer than 5% had type 2 diabetes, inflammatory disease, chronic renal failure, history of ischemic cardiovascular accident, history of stroke, or heart failure.

**Table 1:** Patient characteristics (Full Analysis Set; *N* = 99 patients)

		Overall (*N* = 99)
Age, years	Median (Q1; Q3)	71 (66; 78)
	≤ 70 years, *n* (%)	45 (45.5)
	> 70 years, *n *(%)	54 (54.5)
Age at menopause onset, years	Missing	1
	Median (Q1; Q3)	50 (49; 52)
Age at OP diagnosis, years	Median (Q1; Q3)	66 (61; 74)
BMI, kg/m^2^	Missing	2
	Median (Q1; Q3)	24.3 (21.2; 27.9)
	< 18.5 kg/m^2^, *n* (%)	5 (5.2)
	≥ 18.5 kg/m^2^, *n* (%)	92 (94.8)
*T*-score, reference DXA		
Lumbar	Missing	2
	Median (Q1;Q3)	−2.3 (−2.8; −1.5)
Femoral neck	Missing	3
	Median (Q1;Q3)	−1.6 (−2.3; −1.3)
Hip	Missing	70
	Median (Q1;Q3)	−2.0 (−2.4; −1.3)
Oral BP treatment duration, years, *n* (%)	Missing	10
	Median (Q1; Q3)	2.8 (2.3; 3.5)
	≤ 3 years, *n* (%)	47 (47.5)
	> 3 years, *n* (%)	35 (35.4)
Time between OP diagnosis and oral BP initiation, years	Median (Q1; Q3)	0 (0; 1)
OP fracture before oral BP initiation, *n* (%)	Overall	9 (9.2)
	Spine	4 (4.0)
	Wrist	2 (2.0)
	Femoral neck	1 (1.0)
	Shoulder	1 (1.0)
	Foot	1 (1.0)
OP fracture as reason for 1 st BP prescription	*n* (%)	8 (8.1)
Father/mother OP hip fracture	*n* (%)	11 (11.3)

Full Analysis Set includes enrolled patients who met the study eligibility criteria and had a baseline and follow-up DXA. Summary statistics were calculated for patients with non-missing data

*BMI* body mass index, *BP* bisphosphonate, *OP* osteoporosis/osteoporotic

### Agreement of GPs’ treatment decision with SFR/GRIO treatment guidelines

**Fig. 2:**
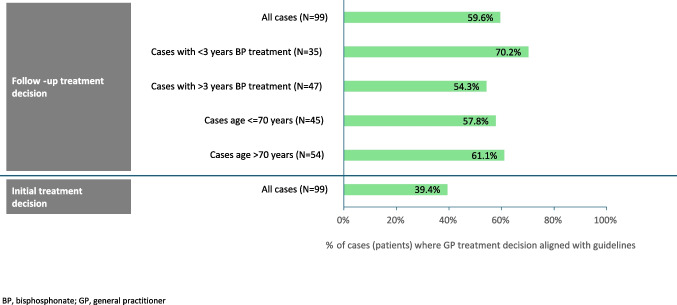
Agreement of treatment decision with treatment guidelines (Full Analysis Set; *N* = 99 patients)—overall and summarized by duration of oral BP treatment and age

Figure 2 summarizes overall agreement of the participating GP treatment decisions with guidelines (as assessed by the first and senior authors). Overall, the participating GPs’ decision to stop, continue or change oral BPs were aligned with recommendations in 59.6% (59/99) of patients. Compared with patients who had been receiving oral BPs for more than 3 years, agreement was higher in patients who had been receiving oral BPs for less than 3 years (70.2% [33/47] vs 54.3% [19/35]). No differences were observed between patients aged ≤ 70 years vs > 70 years (26/45 [57.8%] vs 33/54 [61.1%]. Our concordance with the GPs’ follow-up treatment decisions was weak (kappa coefficient of 0.295).

The GPs’ decision to initiate oral BP treatment was aligned with recommendations in 39.4% (39/99) of patients, with the follow-up decision to stop, change, or continue treatment aligned with recommendations in 71.8% (28/39) of these patients.

### Bone-density parameters used to make treatment decisions

Table 2 summarizes the bone-density parameters used to make follow-up treatment decisions. In most patients, both the baseline and follow-up DXA assessments were used to decide whether to continue, change or stop oral BP treatment (participating GPs, 90.9%; authors, 97.9%), with *T*-scores the most commonly used parameter (participating GPs, 76.8%; authors, 92.9%) and lumbar spine the most commonly used site (participating GPs, 81.8%; authors, 90.9%). Compared with participating GPs, the authors were more likely to use raw BMD values (80.8% versus 35.4%) assessed at the lumbar spine (90.9% versus 81.8%) or femoral neck (89.9% versus 75.8%).

**Table 2:** Bone-density parameters used to make follow-up treatment decisions for *N* = 99 patients in the Full Analysis Set

	Participating GPs (*N* = 99)	Authors (*N* = 99)
DXA, *n* (%)		
Missing	0	5
Initial DXA only	3 (3.0)	1 (1.1)
Follow-up DXA only	6 (6.1)	1 (1.1)
Both	90 (90.9)	92 (97.9)
Parameters, *n* (%)		
BMD *T*-score	76 (76.8)	92 (92.9)
BMD *Z*-score	3 (3.0)	0 (0.0)
BMD values (g/cm^2^)	35 (35.4)	80 (80.8)
Sites, n (%)		
Lumbar spine	81 (81.8)	90 (90.9)
Total hip	19 (19.2)	23 (23.2)
Femoral neck	75 (75.8)	89 (89.9)

Full Analysis Set includes enrolled patients who met the study eligibility criteria and had a baseline and follow-up DXA. Summary statistics calculated for patients with non-missing data

*DXA* dual-energy X-ray absorptiometry, *BMD* bone mineral density

**Fig. 3:**
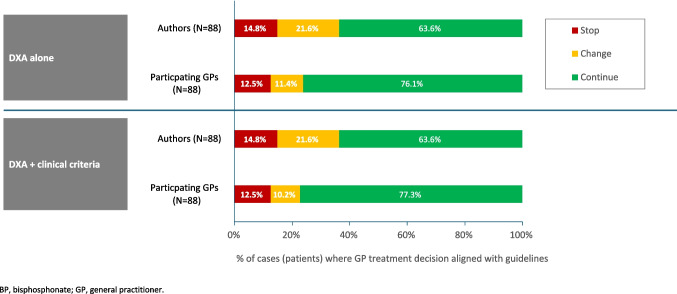
Agreement of decision to continue, change, or stop oral BP treatment summarized by criteria used for *N* = 88 patients in the Full Analysis Set with available data

Figure 3 summarizes the agreement of individual treatment decisions when DXA was used alone or in combination with clinical criteria (data available for *N* = 88 patients). Using DXA alone, the authors would have continued oral BPs in fewer patients than the participating GPs (56/88 [63.6%] vs 67/88 [76.1%]) and discontinued treatment in a similar number of patients (13/88 [14.8%] vs 11/88 [12.5%]). Similar results were observed when DXA was used in combination with clinical criteria.

When changing treatment, and consistent with the participating GPs, the authors would have switched to injectable BPs (participating GPs, 1/10 [10.0%]; authors, 4/19 [21.1%]), or to another therapeutic class (participating GPs, 9/10 [90.0%]; authors, 15/19 [78.9%]). However, the consistency of these decisions between the participating GPs and authors was weak (kappa coefficients < 0.4).

### Changes in BMD between initial and follow-up DXA

Change in BMD between baseline and follow-up DXA are summarized by follow-up treatment decision in Table 3. When the treatment decision was to stop oral BPs, patients experienced a median increase of 0.06 g/cm^2^ at the femoral neck and 0.08 g/cm^2^ at the lumbar spine. Smaller increases were observed in patients for whom the decision was to continue treatment (femoral neck, 0.01; lumbar spine, 0.03) and decreases observed in patients for whom the decision was to switch to another oral BP (femoral neck, −0.06; lumbar spine, −0.02).

**Table 3:** Changes in BMD between initial and follow-up DXA (Ful Analysis Set, *N* = 99 patients)

	Treatment		
	Continued *N* = 77	Changed *N* = 10	Stopped *N* = 12
Femoral neck, median (Q1; Q3)			
Initial BMD (g/cm^2^)	0.78 (0.70; 0.88)	0.79 (0.72; 0.86)	0.65 (0.60; 0.80)
Follow-up BMD, g/cm^2^	0.81 (0.68; 0.91)	0.72 (0.66; 0.78)	0.74 (0.66; 0.88)
Difference, g/cm^2^	0.01 (−0.02; 0.04)	−0.06 (−0.17; −0.01)	0.06 (0.03; 0.09)
Lumbar spine, median (Q1; Q3)			
Initial BMD (g/cm^2^)	0.88 (0.77; 0.97)	0.88 (0.81; 0.94)	0.87 (0.74; 1.08)
Follow-up BMD, g/cm^2^	0.93 (0.82; 0.98)	0.82 (0.73; 1.02)	0.99 (0.82; 1.15)
Difference, g/cm^2^	0.03 (−0.01; 0.07)	−0.02 (−0.07; 0.06)	0.08 (0.04; 0.16)

*DXA* dual-energy X-ray absorptiometry, *BMD* bone mineral density

**Fig. 4:**
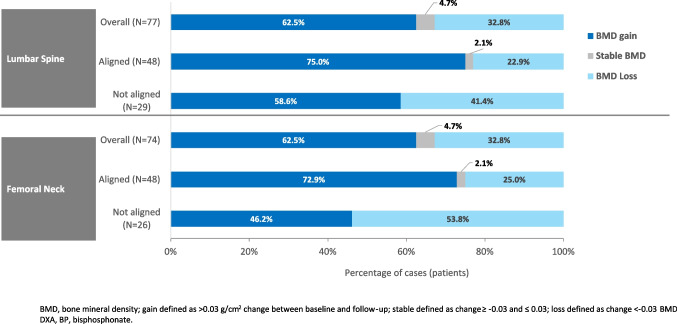
BMD changes between initial and follow-up DXA, summarized by the study investigators’ decision to continue, change, or stop oral BP treatment (patients in the Full Analysis Set with available data)

Figure 4 summarizes the proportion of patients with a gain, loss, or stable BMD by agreement of the participating GPs’ follow-up treatment decision with treatment recommendations. Patients for whom the follow-up treatment decision was aligned with recommendations were more likely to have experienced a BMD gain than patients for whom the decision was not aligned (lumbar spine: 36/48 [75.0%] vs 17/29 [58.6%]; femoral neck, 35/48 [72.9%] vs 12/26 [46.2%]).

## Discussion

While GPs play a central role in the management of PMO, there are widely recognized disparities between clinical practice and evidence-based guidelines [11–13]. Moreover, while DXA is recognized as the gold standard method to measure BMD in postmenopausal women, data on how GPs use DXA results to diagnose and manage osteoporosis is sparse. To the best of our knowledge, ours is the first study to evaluate GPs’ adherence to French treatment guidelines regarding the use of DXA scans to guide treatment decisions for oral BPs.

In our study of women with PMO who had received continuous oral BP treatment for 2–5 years, we assessed the overall decision to stop, change, or continue oral BPs to be aligned with guidelines in 60% of cases. Moreover, we assessed the decision to initiate oral BP treatment was aligned with guidelines in only 40% of cases, with the follow-up decision to stop, change, or continue treatment aligned with recommendations in 72% of these cases.

The “crisis of osteoporosis” is characterized by a major gap between the number of patients that should be treated and the number of patients that are effectively treated. Therefore, it is surprising to note in the present study that 60% of patients received inappropriate anti-osteoporosis treatment. Our data add to a small number of studies from other countries which have reported gaps in physicians’ knowledge and application of guidelines regarding osteoporosis management. Neuner and colleagues reported over one third of treatment recommendations made by German GPs did not match guidelines [14]. A previous study was done by the same group [15] using also vignettes of cases with different levels of risk factors for fracture according to age and the importance of lowering the T-score. The authors concluded that one third of the GPs recommended the initiation of antiosteoporosis treatment for patients whose *T*-score was −1. The POSSIBLE [16] study included a large cohort of European postmenopausal women followed by GPs and who were initiating an antiosteoporosis treatment. 25% of these women had no DXA assessment and no past history of fracture. Eleven percent had osteopenia on DXA and no past history of fracture. Overall, 36% of the population began an antiosteoporosis treatment without any rationale. Our study adds to these previous works in its examination of risk perceptions and overtreatment. These inaccurate physicians’ risk perceptions may play a role in poor targeting of care.

In a study of 330 patients from Scottish general practice, Dhillon et al. reported DXA scanning led to a treatment change in at least 60% of cases [17], which is similar to the current study. Richardson et al. (UK) reported UK GPs lacked confidence to utilize DXA scans to make treatment decisions [18]. In a nationwide survey of US resident physicians, Crandall et al. found knowledge regarding osteoporosis diagnosis and treatment to be poor and concluded that undertreatment of osteoporosis is unlikely to improve without increased physician education [19]. Collectively, these data highlight the urgent need to educate physicians regarding clinical guidelines on the diagnosis and management of osteoporosis. Moreover, when both physicians and their patients have a full understanding of the risk and consequences of osteoporotic fractures, treatment decisions are more likely to result in successful clinical outcomes [13].

Data regarding the follow-up of osteoporotic treatments in French primary care is also sparse [7]. For PMO, national guidelines advise a quasi-systematic treatment for patients with prior fracture and, in the absence of an incident fracture, treatment is initiated in patients with a *T*-score ≤ −3 [5, 7]. In the current study, we assessed initiation of oBP treatment to be unjustified in 60% of cases. Neuner et al. reported more than one third of physicians recommended treatment to patients not meeting guideline-recommended treatment thresholds [15]. The women in our study were at low risk of fractures, as shown by only 9% having a history of prior fracture, most (84%) having a *T*-score > −2.5, and few having a BMI < 18.5 kg/m^2^ or diseases or treatments known to induce osteoporosis.

From over 19,000 GPs invited to participate in our study, only 23 accepted and enrolled patients. This very low response rate may limit the generalizability of our data, which should be interpreted with caution. Moreover, this low response rate indicates an overall lack of interest in osteoporosis across primary care and a lack of knowledge regarding the clinical and economic consequences of osteoporotic fractures. In addition, the GPs who participated in this study were theoretically aware of osteoporotic disease, which could bias agreement with clinical recommendations. Participating physicians may have changed their clinical practice after being informed of the study objective, which could also bias the results.

Also, in the aim to improve recruitment, we chose criteria not too stringent regarding the maximum delay (i.e., 2 years) between the first DXA assessment and the beginning of treatment by oBP. However, this relatively long delay could constitute a weakness of the present study.

## Conclusions

Our study suggests insufficient knowledge and application of recommendations for initiation and follow-up of oral BP treatment among GPs. While we assessed their decisions to stop, continue, or change oral BP treatment based on DXA-assessed BMD aligned with French guidelines in 60% of cases, the decision to initiate oral BPs was aligned in only 39% of patients. These data add to the growing body of evidence highlighting the urgent need to strengthen GPs’ knowledge and understanding of treatment guidelines for OP; specifically, the use of bone densitometry in patient follow-up, and, more generally, in the management and treatment of osteoporosis.
